# An Old Mechanism, Imitation, Geared for Socio-Material Knowing in a “Day in the Life” of First Graders

**DOI:** 10.3389/fpsyg.2020.00177

**Published:** 2020-02-14

**Authors:** Giuliana Pinto, Catherine Ann Cameron, Monica Toselli

**Affiliations:** ^1^University of Florence, Florence, Italy; ^2^University of British Columbia, Vancouver, BC, Canada

**Keywords:** sociomateriality, learning, imitation, artifacts, children’s daily life

## Abstract

This paper adopts sociomateriality as a theoretical lens to further our understanding of how imitation acts to support the use of objects, and in doing so, constitutes a sociomaterial practice. Within a sociomaterial perspective we aimed to perform the analysis of imitation as a powerful way to learn how to use objects embedded into the practices within which the objects are constituted. The contribution of this approach is illustrated using the findings of the application of the quasi-ecological *Day in the Life* (*DITL*) methodology to the everyday lives of two 6-year-old children. Within a case-study frame, we traced the children’s imitation behaviors focused on the use of objects during an entire day of their life, the various people and practices with which they were associated, the multiple sociomaterial configurations that the objects assume, and the social and material consequences of their use. Imitation appears to be is a complex activity, involving multiple stakeholders who interact in order to facilitate the understanding of various artifacts across diverse knowledge domains, and enhance their interpretive flexibility across communities of practice.

## Introduction

The meaning of socio-materiality that we adhere to in our research pertains to the way by which a culture encourages its members to interact with material objects. From using forks to eat, to maneuvering high-tech gadgets of modern times, humans are adept in swiftly learning to use a wide range of tools in their daily lives. Mastery of “tool use” implies a progression from learning to act “on” objects to learning to act “with” objects. Among the multiple resources that aid the learning process, the most important are social interactions. How particular modes of interaction are socialized, acquired and internalized by children is worthy of study.

We believe that the use of an object, from a most normative, to a more atypical, personally and socially manipulated useage is transmitted through imitation. Imitation is a powerful mechanism that stimulates the use of a material object, even if it is not the only one, and there are obvious affordances of an object itself. Imitation is a traditional learning and communication tool, long ago identified from (Thorndike, 1898, p. 50) as: “doing an act by seeing it done by someone else,” but much debate has subsequently addressed and investigated this mechanism.

In this research we offer an operational definition, along with introducing certain theoretical concepts that represent imitative behavior. Moreover, different mechanisms underlying imitative behaviors and their different functions in use of material objects can readily be identified. Extensive research (following Meltzoff and Moore, 1977; Gopnik et al., 2000) has addressed infants’ early imitation of motor imitation of adults in controlled experimental settings. Alternatively, an ecological approach to document and explore spontaneous imitative behaviors when objects are involved can be used to illustrate how socio-materiality is shared in childhood by imitation. Transmission of the use of an object corresponds to the transmission of its meaning, value and relational function. The interaction of this learning and communication can trigger the social use of an object.

From early pretend play onward, when a 2-year-old infant “turns objects into symbols”, as Rakoczy et al. (2005) assume, objects acquire a metarepresentational role. “The development of understanding symbolic actions with objects, we claim, is best considered as part of children’s developing social understanding more generally, and the development of performing symbolic actions with objects is most fruitfully viewed as a process of cultural learning, based on children’s nascent understanding of intentional action and on cultural scaffolding” (p. 69).

This cultural learning goes on in every form of interaction with objects performed in front of someone, and can transmit the practical as well as symbolical meaning of objects.

The concept of *guided participation* described by Rogoff (2003) offers instances where the interchange of the meaning of actions, objects, tools are obtained by imitation. The adult partner displays the use of an object in front of a child, thus demonstrating its meaning.

## The Methodological Challenge: The “Day in the Life” (*DITL*) Lens

An examination of the phenomena involved in the induction of socio-materiality via imitation requires methodological choices: First of all, it is necessary to capture as intricately as possible the sequence of the model actions and those of replication: only in their sequence in time, in fact, does their imitative nature make sense and sanction their interpretation. It is then necessary to be able to place such actions in the context in which they take place, accessing a picture as rich as possible in detail, in terms of setting, rules and rhythms, other actors and characters. In this effort we have applied an innovative quasi-ecological methodology, the *DITL* method, originally developed by Gillen and Cameron (2010) and Gillen et al. (2007), whereby the investigators audio-visually record to be able afterward to observe, document, and explore the everyday transactions of young children *in situ* during one specific day which has been over the years confirmed to have both ecological and heuristic validity.

The original methodology has been applied to developmentally different aspects, allowing the researcher to deepen, in socio-cultural perspectives how children comprehend and co-construct (for instance) their symbolic and literacy abilities (Pinto et al., 2008, 2011, 2015), their affective experiences in the use of domestic spaces and their meanings of cultural values for instance (Cameron et al., 2014b; Gillen and Cameron, 2017).

To apply the *DITL* methodology to questions regarding children in the transition between exclusive home- to include formal schooling (Marsico et al., 2013), *DITL* researchers are obliged to adapt the methodology to accommodate the increase of ecological niches across which the children and their families transit through as they enter the school door (Bronfenbrenner, 1986; Sameroff, 2010). This involved the engagement of school authorities, of educational administrators and teachers, of community members including dance, art, swimming and music teachers, youth leaders and neighbors (Cameron and Hunt, 2018). As a result we have had access by audio-visually recording of the constant flow of actions and interactions that accompany and mark the passing of a child’s day from his awakening to his falling asleep at night, through the various contexts (family, school) and with the various partners from time to time, from family, teachers, friends, and neighbors.

Such an approach as that involving the qualitative methods of the *Day in the Life* (*DITL*) procedures provides an efficacious avenue for this sort of dynamic, multifocal, culturally sensitive discovery (cf. Cameron and Pinto, 2020). Moreover following children for just 1 day during their first year of schooling, at home and at school shows the interaction, the zones of overlapping, both contributing in different but sometimes also common pathways to a child’s knowing in every domain, from the strictly social, to the material. Liminality of education, the importance of border zones (Valsiner, 2013), starting formal schooling is a crucial event for young children and their families. How well children negotiate this transition is important, as it affects their long-term academic outcomes (Dockett et al., 2010); this process can be thought of in ecological terms: As children move from home to other learning environments, these environments become increasingly important to their development and the intersection of family and school is a crucial third locus for development and education (Sameroff, 2010).

## Imitation in a *DITL* of Children in Transition to School

Ecological observation of the everyday events of typically developing children, potentiated by a audiovisual methodology offers us a uniquely valuable lens through which to capture the interesting and underestimated form of early social learning: imitation. As many sociologists (cf. Berger and Luckman, 1967) presume, the meaning of life is concentrated in the experience of daily life events. Let us therefore define the meaning we attribute to imitation, as it is a concept worthy of careful consideration. Paulus (2011) stressed that a behavioral definition of imitation can be helpful. While broadly encompassing, this definition can also be wholly sharable.

“… imitation is designated in all the instances when infants show the same behavior a model has performed in front of them and as a consequence of the particular action the model has performed (and not as a consequence of any other behavior). We can clearly see that, assuming this analysis, the word imitation comes with some assumptions about the imitative behavior. Imitation is then used when a relation between two behaviors is assumed in which a second behavior is sufficiently similar and causally connected to a first behavior without concretely specifying the mechanism which subserved it in detail” (Paulus, 2011, p. 850).

Consequently:

“…when observing infants in daily life, we hardly ever know by which mechanism their imitative behavior actually was subserved, especially when we are aware that this might also change from situation to situation so that the same behavior in one situation could be caused by reading the intention of others and in another situation by emulation or mimicry. We only can state at a behavioral level that infants imitated somebody” (Paulus, 2011, p. 852).

Inasmuch as it would be possible to consider in imitative behavior every enactment that somehow reproduces an earlier one, including when the immediate presentation by the model is not in sight, delayed imitation might be considered. But considering examples of extended delays in imitation would also divert our efforts to identify imitation solely by visual support what was captured by in the audio-visual record. Thus, we do not consider in our research delayed imitation behavior, as they could misdirect our aims.

Having focused our attention on motor repetition to identify imitation, we have subscribed to Byrne’s assumption that “imitation is magical” (Byrne, 2005, p. 225), as imitative behaviors by definition are just those behaviors that are not causally explained by the more common mechanisms of reinforcement. Accordingly, we hypothesize that this mechanism works very frequently in children’s everyday lives (Toselli et al., 2018) and we will explore partners and settings that enhance using material objects in diverse ways, by imitation.

## Methodology

Two Italian children were video-recorded during an entire *DITL* (cf., Toselli et al., 2018) each for ten-hour-long continuous episodes, across home (about 5 h) and school engagement. During a *DITL*, a child is carefully filmed from the time s/he repetition. In advance of the filming, the primary socializers (in the home: the parents; in the school: the teachers) are interviewed and they collaborate on providing access to a usual *Day*’s events, interactions and contexts of participation. The measures to reduce the interference constituted by the presence of the observer, consisted not only in the specific training and experience of the observer to that particular observational method, but also in the preliminary knowledge and presentation of the observers to the family in the days preceding the one chosen for the registration of the overall day. While the child is filmed, a note-taker records and maps contextual and cultural information, important in the interpretation the transactions viewed on film. No assumption of typicality in this cultural project is made. Our analysis simply draws upon this recorded corpus of naturalistic interactions between our participants, their adult interlocutors and their siblings and peers, selecting those transactions involving imitation of interactions with material objects. Our two participating first graders were observed while they successfully navigated 1 day during their first year in primary school, following their preschool experiences. They were identified as healthy and thriving children in the framework of a positive psychological research initiative. The videotaped material inspired this investigation of motor imitation pertaining to the use of objects, observable by the careful inspection of the visual data recorded by the videotapes. We therefore identified every motor sequence that was repeated or inspired by each of the two participants under study. On the corpus of video recordings relating to the entire day of the two participants, two independent judges, members of the research team, carried out a visual examination of the material with the task of detecting all the episodes in which motor imitation behavior as previously defined was present, creating a list. The comparison between the choices made by the two judges revealed a 100% agreement. The episodes reported are therefore all those identified with the agreement of the two judges. The interpretation of the events thus identified was carried out jointly by the authors, and elaborated in the form of a discussion before articulating the commentary of each of the episodes reported below, with a cyclically inductive and deductive approach, from data to theory and vice versa. The selected imitation situations were extracted from the stream of the daily interactions, by two observers, independently inspecting the 10 h of video-recordings of each child’s “*Day.”* One videographer followed and filmed each child throughout their day while a second researcher took careful contextual notes. There was minimal verbal communication between the researchers and participants during the recording of the *day(s)*. Rather, semi-structured interviews were conducted before and after the data collection day. Before hand, participants (child, parents, siblings, teachers, and peers) were fully appraised in advance as to the procedures they were to expect, and afterward, participants, their families and teachers were shown a half-hour compilation of filmed clips of the full filmed day and asked to comment in general on the selected clips and specifically, as to whether they were somewhat representative of the child’s daily life.

## Participants

Our two Italian first graders are a boy, 6,7 year-old Martino, and Sara, a 7,1 year-old girl, both attended the same class in a school in a suburban area on the outskirts of a city in Central Italy, characterized by a medium socio-economic level. In Italy, most children (approximately 99%) are enrolled in state schools, which thus provides a representative cross section of the Italian population among kindergartners and primary school students. the Italian population is characterized by a very low mobility, and children tend to attend schools in the same neighborhood.

The research was performed in the first year of a State primary school, attended by children between 6 and 7 years of age. The aim of this level, compulsory, in the education system is to provide pupils with basic learning and the basic tools of active citizenship. Primary education is divided, for teaching purposes only, into a first year, linked to pre-primary school, followed by a further two levels of 2 years each. In primary schools, children, according to their age, are organized into groups called “classes.” The class where we worked with 22 pupils and, adopting the weekly school timetable of 30 h, had only one teacher, a generalist supported by an English language teacher. The standard school day consists of a total of 7 h, during which from 8.30 a.m. to 1 p.m. and from 2.30 p.m. to 4.30 p.m. the teaching activity takes place. While from 1 p.m. to 2.30 p.m. the meal is eaten inside the school structure in the presence of special staff.

Children in the Italian educational system typically start kindergarten at age three and finish when they are five. Then, children enroll in primary school when they are 6 years old. Moreover, in Italian schools, children are exposed to formal reading as soon as primary school begins. By contrast, the national curriculum for kindergarten does not include the formal teaching of reading and writing. According to national guidelines (Law n. 254 of November 16, 2012), first-grade children are expected to learn the instrumental level of the written language (reading and spelling) and the basics of mathematics (arithmetic, logic, geometry, and measuring). Teachers-students ratios are 2:28 in kindergartner and 1:25 in primary school.

The two participants, Sara and Martino, also share cultural environments in terms of the resources available to their families, and the character of the communities in which children live, including the economic climate and accessibility of appropriate services. The local community whose population is 95% Italian, is predominately middle-class, with most parents having had at least some college education.

Sara is attending the first year of a public Italian primary school, and she and her older brother live with their parents, who are both professionally employed. The family lives in a detached home, within walking distance of the school. Martino, also lives with his parents, both professionals, and his younger sister in an apartment with a garden in a renovated farmhouse. He also goes to school on foot, generally accompanied by his father.

As part of our ethical procedures, school authorities and parents offered informed consent for participation in the study and the children afforded active assent.

## Data Analysis

All instances during which partners show the same motor behavior (addressing an object) that a model has performed in front of them, that is, doing an act after seeing it done were identified. The participants can be either the copier or the model, as it is common in this age old. As a note of caution, a single situational description cannot provide conclusive evidence to the reader of what happened in the dynamic flow of the video recording. Thus the instances were interpreted to identify the various forms of cultural learning that took shape when children, at the beginning of primary school are ubiquitously imitating the use of culturally significant objects. Our expectation is that imitation can lead to the learning of different modalities and nuances about the functions and rules with which objects can be used (i.e. their pragmatics), depending on the model and context. Across diverse social environments, that differ in the extent and the conditions with which children are engaged by significant others.

## Findings

There were a total of 23 separate imitative episodes in the day-long video-recorded material pertaining to Sara and 30 imitations in the video-recoding of Martino’s “*Day.*”

Among these imitations, we identified some 8 of them that involved the use of objects: 3 for Sara and 5 for Martino.

We first present the 3 imitations where a normative use of objects is demonstrated by the model and subsequently executed by the imitating partner and then, we describe the 5 more original and subjectively laden imitative use of objects. Some imitative scripts involve adults as well as peers. Episodes occurred at home as well as at school.

(1) The most typical episode where a normative use of objects is shown involved an adult. Sara is engaged in a craft activity with her mother at home, creating a bracelet with glass beads: Mother shows her how to craft the bracelet. Her mother offers a model for enacting a behavior that is to be acquired by her child. Sara engages in an imitative activity during a session of what Rogoff (2003) would refer to as guided participation. The girl waits to see her mother’s pearl threading and immediately imitates the simple procedure, then proceeds by herself, replicating the newly learned behavior. While proceeding with her work she asks her mother when to stop stringing a strand of beads, the mother shows her how to measure, around the child’s arm, the necessary length of the bracelet, and Sara, after adding some beads, uses the same measuring procedure.

(2) In peer tutoring, however, reciprocity emerges. After school, at home, Martino and a schoolmate, who accompanied Martino home from school, are playing football. One of them knows how to advance the ball with his head, the other, with his foot. They reciprocally enact for one another the motor performance and model for each other. Reciprocity is a hallmark of imitation between peers, and less frequently, in exchanges with adults, who most characteristically only deploy reciprocity in early interactions with very young children. Playing football represents the social and cultural contexts in which these children live: Socio-cultural variations depend not only upon the attitudes of parents, teachers, and society in general, but also on such variables as the amount of play space and time that is available to children Martino and his schoolmates help each other in an imitative activity that produces some physical acts to pursue specific goals according to codified rules.

(3) Older brothers are also typical models for the canonical use of objects by younger siblings. During breakfast, while still at home, Martino’s little sister, even if without the specific need to wipe her mouth, as soon as Martino has wiped his mouth, she immediately imitates him, she too uses a paper towel that is on the breakfast table. Table manners evolve within cultures, varying widely between different cultures and countries, and they exert a useful function in intercultural adaptation. To master fundamental knowledge of table manners in a culture enhances intercultural communication awareness and intercultural adaptation. In their mutability from one culture to another, they constitute a good example of an “opaque rule,” that is, highly conventional, arbitrary and unpredictable. To learn good manners at the table, imitation appears to be a powerful, if not the only, aid.

The use of objects can also present indications of creativity, introducing varied interactions with them, which can also convoy a personal communicative message about the object’s meaning of its use in a relationship.

(1) A creative, playful activity can arise: When Sara plays with her shoe laces, as if they were musical instrument strings, two schoolmates discover this new behavior and subsequently play with their own shoe laces as strings, demonstrating a kind of a diversified imitation. Learning requires an interactive balance of gaining the facts and skills required by the culture and making information one’s own. We are shown, in this imitative istance, how children enact this interactive cycle, that helps them to understand the use of objects in an intrinsically motivating way.

(2) Still at school, during the common meal in the canteen, we detected an immediate and exact imitation by Martino, observing and then replicating a schoolmate’s dunking a cracker in his glass of water before eating it. This is an original use in our food culture, where crackers are not softened in cold water, but rather in other, usually hot, beverages! Interaction with peers in less rigid situations than academic routines, such as those allowed during school meals shared together, offers valuable opportunities to children to act and behave like people they know. Freedom to use “traditional” objects encourages creative expression of ideas and understandings.

(3) Again at school, during the school meal, we have an imitation of a procedure for managing the precarious positioning of a full water pitcher, on the edge of the dining table, beside a glass, also full of water. This behavior is reenacted by a schoolmate who follows Martino and asks him for explicit instructions and demonstrations in order to get the same result as the model. This more perilous activity, which could produce a mess on the table, clearly shows the atypical use of objects, introducing in it a prohibited aspect, if noticed by the teachers. Martino and his companions show that they know how to use hybrid spaces to navigate between rules and invention, experimenting with the borderline boundaries of the conventional use of objects.

(4) During a recreation period at school, at a time that is not typically devoted to instructional learning, such as during formal lessons, we observed a more aggressive kind of imitative behavior, involving a materially valued object. A group of children are looking at a sticker album placed on the ground out in the garden. Martino steps onto the album with shoed feet, interrupting his schoolmates’ activity. One of his admirers immediately steps in the same way onto the album. Culturally representational materials help children understand the social and ethnic values of their communities. Such social contexts provide them with an arena for refining their social skills also through the conception and imitation of transgressive behaviors, in which the cultural value of objects is challenged.

(5) At home Sara is playing in the room she shares with her older brother. The atmosphere is playful and somehow conflictful because the two children are debating about who is the owner of various plush animal toys that are in a basket. Sara begins throwing the toys in the air and also at her brother. The brother immediately recognizes the provocative value of this activity and does the same with the objects, especially those that he believes to be property of his sister. In this interaction, children exploit the opportunity to move freely from one area to another and to engage in recreational activities in spaces of the house that are not continuously and directly under the control of adults. Imitation-based learning activities provide multiple ways for children to learn to use complex, challenging, and varied materials.

## Discussion

This paper takes up the socio-material perspectives as an avenue for understanding the role of imitation in learning to use objects in family and school practices, assuming that the range between informal and formal learning can be conceptualized as a continuum.

Our visual theoretical approach and its consequential methodologies deployed within the *DITL* sociocultural framework proved to be effective, deepening our understanding of collaborative construction of the use of objects during a critical life transition namely, when they first gain significant learning experiences outside the home, and specifically in a formal school setting (Cameron et al., 2014a). Using examples of children’s acts of imitation during their filmed *Day*, we documented how school and home are contexts populated by significant others whose knowledge and practices offer to the child multiple models learning true imitation. We also discovered how frequent, multifaceted and powerful imitation exists across contexts and partners and how children use imitation in a collaborative and communicative manner as a powerful meaning-making tool. The procedure adopted in the *DITL* research allowed us to provide rich information regarding the thinking, talking, and social interaction that naturally occurred “around” imitations the child perform across the continuities and discontinuities that characterize the way in which objects are used in the various systemic practices.

We discovered from our observations that imitation in the motor use of objects resides in the commonplace of children’s every day lives. These uses transmit a normative and sometimes a more creative, subjectively and emotionally laden use of objects.

In this double context of school and home we noticed that whether our recordings pertained school- or home-time, is was mostly when the children interacted freely, that the guided participation with the use of objects was enacted. Our participants demonstrated both *agency and communitarianism* in this period of their lives, just as we reported of our young Canadian participants (Dmytro et al., 2014), characterized by many new and often conflicting demands for competence. Formal schooling classically pertains to the immaterial, abstract world of learning and explicitly encourages, in a collaborative framework, imitation by pupils, aimed at academic tasks and hewing to the rules of the classroom culture. But, as far as we have been able to observe on the school day of Sara and Martino, it is not the objects and their utilities that are prompting imitation, but rather the practical navigation of school rules and the pragmatics of addressing assigned academic tasks. It is in free time that, at this age, in transactions with adults as well as peers, knowledge of the material world is tested, explored and acquired. The family, less focused on explicit teaching, more frequently uses demonstrations of what the child needs to imitate to use instruments, as was the case of Sara and her mother crafting the bracelet. Differences emerge also between partners: Adults can be imitated, but they seldom-to-never imitate children, while with peers reciprocity is more common, as in Martino’s dangerous play with the pitcher full of water. We discover in these first graders’ lives the wide role expressed as models by peers as to the material world. It is quite obvious that the amusing, trasgressive role of managing objects is particularly shared by peers!

Focusing on children’s imitative behaviors we aimed further to enrich our understanding of the young child’s perspectives. Adopting a visual methodology we had the opportunity not only listening to verbal communication, but also checking the child’s different forms of expressing non-verbal emotions, meanings and sense of everyday events. When researchers attempt to capture the lived experiences and sense of everyday events from the young child’s perspective through the use of visual methodologies, they acknowledge the unspoken voice of the child which is always present in their affective, active engagement with others and the environment (Quiones, 2014).

Social scientific research also does not yet reveal a deep enough understanding nor does it apply ready opportunities for exploring such mechanisms as imitation that we share with so many species and, living beings, beyond childhood and across the life span. This paper has argued strongly for legitimizing imitation as an appropriate learning tool in schools and other educational settings. We tend to be blind to the all-pervasive constructivist imitative nature of human beings and particularly young children, and unaware of its implications for parenting and educating. In their everyday lives, children are busy observing those around them and interpreting the world accordingly: Imitation plays a crucial role in the transmission and maintainance of relevant cultural knowledge, specially suited for the demand characteristics of cultural forms (as the symbolic tools and instruments), whose causal, functional, or intentional nature is cognitively opaque to the learner (Gergely and Csibra, 2006) who can therefore only acquire them through imitative actions.

## Data Availability Statement

The datasets generated for this study are available on request to the corresponding author.

## Ethics Statement

The study involved human participants and was approved by the Ethical Review Committee of the Department of Psychology, University of Florence. School authorities offered informed consent, parents provided written informed consent for the children’s participation, and the child-participants afforded active assent.

## Author Contributions

All authors listed have made a substantial, direct and intellectual contribution to the work, and approved it for publication.

## Conflict of Interest

The authors declare that the research was conducted in the absence of any commercial or financial relationships that could be construed as a potential conflict of interest.
